# The Role of Hypoxia in Improving the Therapeutic Potential of Mesenchymal Stromal Cells. A Comparative Study From Healthy Lung and Congenital Pulmonary Airway Malformations in Infants

**DOI:** 10.3389/fbioe.2022.868486

**Published:** 2022-06-14

**Authors:** Serena Silvestro, Francesca Diomede, Luigi Chiricosta, Valeria Domenica Zingale, Guya Diletta Marconi, Jacopo Pizzicannella, Andrea Valeri, Maria Antonietta Avanzini, Valeria Calcaterra, Gloria Pelizzo, Emanuela Mazzon

**Affiliations:** ^1^ IRCCS Centro Neurolesi “Bonino-Pulejo”, Messina, Italy; ^2^ Department of Innovative Technologies in Medicine and Dentistry, University “G. D’Annunzio” Chieti-Pescara, Chieti, Italy; ^3^ Department of Medical, Oral and Biotechnological Sciences, University “G. D’Annunzio” Chieti-Pescara, Chieti, Italy; ^4^ Ss. Annunziata” Hospital, Chieti, Italy; ^5^ Cell Factory, Pediatric Hematology Oncology Unit, Fondazione IRCCS Policlinico San Matteo, Pavia, Italy; ^6^ Pediatrics and Adolescentology Unit, Department of Internal Medicine, University of Pavia, Pavia, Italy; ^7^ Pediatric Department, Children’s Hospital “Vittore Buzzi”, Milano, Italy; ^8^ Pediatric Surgery Department, Children’s Hospital “Vittore Buzzi”, Milano, Italy; ^9^ Department of Biomedical and Clinical Sciences-L. Sacco, University of Milan, Milan, Italy

**Keywords:** hypoxia, mesenchymal stromal cells, lung, congenital pulmonary airway malformations, transcriptomic analysis

## Abstract

Mesenchymal stromal cells (MSCs) play an important role in the field of regenerative medicine thanks to their immunomodulatory properties and their ability to secrete paracrine factors. The use of MSCs has also been tested in children with congenital lung diseases inducing fibrosis and a decrease in lung function. Congenital malformations of the pulmonary airways (CPAM) are the most frequently encountered lung lesion that results from defects in early development of airways. Despite the beneficial properties of MSCs, interventions aimed at improving the outcome of cell therapy are needed. Hypoxia may be an approach aimed to ameliorate the therapeutic potential of MSCs. In this regard, we evaluated the transcriptomic profile of MSCs collected from pediatric patients with CPAM, analyzing similarities and differences between healthy tissue (MSCs-lung) and cystic tissue (MSCs-CPAM) both in normoxia and in cells preconditioned with hypoxia (0.2%) for 24 h. Study results showed that hypoxia induces cell cycle activation, increasing in such a way the cell proliferation ability, and enhancing cell anaerobic metabolism in both MSCs-lung and MSCs-CPAM-lung. Additionally, hypoxia downregulated several pro-apoptotic genes preserving MSCs from apoptosis and, at the same time, improving their viability in both comparisons. Finally, data obtained indicates that hypoxia leads to a greater expression of genes involved in the regulation of the cytoskeleton in MSCs-lung than MSCs-CPAM.

## Introduction

Cell therapy is an important field that sees use in tissue engineering and regenerative medicine (Huang et al., 2009). Human mesenchymal stromal cells (MSCs) possess the ability to self-renewal and multilinear differentiation (Minguell et al., 2001; Pittenger and Martin, 2004) and have been isolated from different tissues such as fat, bones (Pittenger and Martin, 2004), bone marrow (Minguell et al., 2001), teeth (Huang et al., 2009), amniotic fluid (Kaviani et al., 2003), and urine (Chun et al., 2012). Thanks to their immunomodulatory properties and their ability to secrete several paracrine factors, they have been used for the treatment of cardiovascular diseases (Thakker and Yang, 2014), nerve injuries (Dadon-Nachum et al., 2011), bone regeneration (Fan et al., 2015) and respiratory disorders (Lou et al., 2021). Furthermore, the use of cell therapy with MSCs was also tested in children with congenital lung diseases (Pelizzo et al., 2020; Tong et al., 2021).

As reported, repeated administration of allogeneic MSCs improved respiratory conditions in children with interstitial lung disease. In particular, in the lung tissue, the regenerative processes are promoted by a pool of MSCs which would appear to be involved in the regeneration and architecture of tissues processes. In conditions such as congenital malformations of the pulmonary airways (CPAM), the therapeutic potential of MSCs ha also been proposed (Pelizzo et al., 2021).

CPAM are congenital pulmonary anomalies histologically characterized by the presence of multiple cysts located in the lung parenchyma. Although considered a rare disease, it is estimated to affect between one in 25,000 and 35,000 live births (Laberge et al., 2001; Duncombe et al., 2002). These lung lesions are the result of embryological damage in the early gestation period causing hyperproliferation and terminal bronchioles dilatation. The absence of normal alveoli induces pulmonary compression and hypoplasia and in some cases also a displacement of the mediastinum (Stocker et al., 1977; Stocker, 2009). CPAM are classified into 5 types according to the size and lesion location. Among these the most recurrent lesions are the type 1 lesion, which occurs in 60–70% of cases with intercommunicating cysts >2 cm, and CPAM types 2 in 15–20% of cases with cystic lesions <2 cm (MacSweeney et al., 2003). It can be asymptomatic or be responsible for recurrent infections or symptoms related to compression of the airways. To date, treatment in symptomatic patients includes surgical therapy with excision of the lesion (Leblanc et al., 2017). In the asymptomatic forms, indeed, the risk of developing infections and the potential development of tumors seem to support resection in childhood (Aslan et al., 2006; Adzick, 2009; Pelizzo et al., 2009; Peters et al., 2013; Gajewska-Knapik and Impey, 2015). MSCs have a crucial role in the microenvironment and regulation of tumor survival, growth, and progression (Pelizzo et al., 2017). However, MSCs could be useful for the lung regenerative process after surgical treatment of congenital pulmonary lesions.

For this reason, interventions aimed at improving the regenerative capacities of MSCs are needed. Several studies reported that the use of growth factors or exposure to hypoxic conditions improves the properties of MSCs (Khan et al., 2011; Fan et al., 2015). Oxygen (O_2_) is a substrate needed by cells for energy production and cell metabolism (Wang et al., 2005). Cell proliferation and differentiation are biological processes regulated by O_2_. Hypoxia preconditioning has been proposed as an approach to improve the therapeutic potential of MSCs; in fact, it has been seen that short-term exposure to hypoxia has an empowering effect leading to increased cell migration and improved proliferation, survival, differentiation and paracrine activities of MSCs (Annabi et al., 2003; Yang et al., 2022).

In this context, MSCs were harvested from infants after surgery for CPAM’s lesions. Healthy lung tissue (MSCs-lung) and cystic tissue (MSCs-CPAM) were considered. In order to investigate the effect of O_2_ concentration, MSCs-lung and MSCs-CPAM were exposed to *in vitro* normoxia or hypoxia (0.2% O_2_) condition for 24 h. Subsequently, analogies and differences in transcriptomic profiles between MSCs-lung or MSCs-CPAM in normoxia against hypoxia were analyzed.

## Materials and Methods

### Patients

Two male infants with diagnosed CPAM were admitted to the surgical ward for elective surgery including lobectomy. Microscopically, the lesion was diagnosed as CPAM type II by Stocker’s classification. With parental consent, a portion of the lung tissue intended for histological analysis was used for the expansion of MSCs. Two samples were obtained respectively from the “healthy” tissue (called MSCs-lung) and from congenital lung tissue (called MSCs-CPAM). The study was performed according to the Declaration of Helsinki and with the approval of the Institutional Review Board of the “G. Di Cristina” (registration number 87 Civico 2017). Written informed consent was obtained by the parents and/or legal guardian of each infant after receiving information on the study.

### Cell Isolation, Culture and Characterization

The MSCs were obtained from pulmonary tissue of two male infants diagnosed with CPAM and submitted to elective surgery, as previously reported (Pelizzo et al., 2017). The cells were expanded until passage 2. Subsequently, the cells were seeded at 2,5 × 10^6^ cells in each Petri dish (150 × 25 mm) and kept in culture with MSCBM (Lonza, Basel, Switzerland) culture medium for 24 h inside an incubator at 37°C with a humidified atmosphere at 5% CO_2_ in the air before hypoxic treatment.

The cells were characterized as previously reported for:

- proliferative capacity defined as calculated cell count (CCC),
- immunophenotype by flow-cytometry using monoclonal antibodies specific for CD73, CD34, CD90, CD14, CD45, CD31, CD105 class I and class II- HLA (Beckman coulter Milan, Italy),
- osteogenic and adipogenic differentation capacity was evaluated by *in vitro* adding specific stimuli. In particular, for osteogenic differentation 10−7 M dexamethasone, 50 mg/mL L-ascorbic acid and 5 mM ß-glycerol phosphate were added to α-MEM 10% FBS (Euroclone, Milan, Italy), for adipogenic differentiation 100 mg/ml insulin, 50 mM isobutylmethylxanthine, 0.5 mM indomethacin were also added to culture medium. All reagents were from Sigma-Aldrich (Milan, Italy).
- The capability to enter senescence was evaluated maintaining cells in culture until the number of detached cells was ≤ to the number of plated cells.
- The immunomodulatory capacity was assesed at different ratio (1:2-1:20-1:200) on healthy donor peripheral blood mononuclerar cells (PBMC) activated with phytohemagglutinin (PHA).

### Hypoxic Treatment

The cultured cells of both MSC-lung and MSCs-CPAM were seeded in a 6-multiwell plate with a density of 8 × 10^4^ for each well. After seeding cells were maintained for 24 h in the standard medium MSCBM (Lonza) in incubator at 37°C with a humidified atmosphere at 5% CO_2_. Then cultured cells of MSC-lung and MSCs-CPAM were subjected to hypoxic treatment by inserting the plates into the ProOx Model P110 (BioSpherix, New York, NY, United States) hypoxia chamber for 24 h at 0.2% of hypoxia. Cells were observed under inverted light microscopy (Leica Microsystem, Milan, Italy) to evaluate the morphological features in normoxic and hypoxic conditions. Cultured cells of MSC-lung and MSCs-CPAM were maintained in incubator with normoxic condition and used as control cells.

### RNA Extraction

RNA isolation was performed using the Total Exosome RNA and Protein Isolation Kit (catalog # 4478545; Thermo Scientific, Rockford, IL, United States) following the manufacturer’s instruction. The RNA quality and concentration were measured using Eppendorf BioSpectrometer fluorescence. TruSeq RNA Exome protocol (Illumina, San Diego, CA, United States) was used for library preparation. 3 batches were analyzed by RNA sequencing. Concisely, each sample RNA extracted was fragmented at 94 C for 8 min and then was synthesized the first strand of cDNA using the SuperScript II Reverse Transcriptase (Invitrogen, Milan, Italy). Subsequently, the second strand of cDNA was synthesized and purified using AMPure XP beads (Beckman Coulter, Brea, CA, United States). In the following step, in order to allow the adaptor ligation, the 3′ ends of the cDNA were adenylated and then was performed the indexing adapter ligation. The library was purified with AMPure XP beads. In order to enrich those fragments of DNA that have adaptors on both ends, and also to increase the quantity of library DNA a first PCR amplification step was carried out. The library has been validated through the Agilent Technologies 2,100 Bioanalyzer and then, 200 ng of each DNA library were combined for performing the first hybridization step. Magnetic beads coated with streptavidin were used to capture probes hybridized to the target regions, in order to eliminate nonspecific binding. After the enriched libraries were eluted from the beads, the second cycle of hybridization was performed, in order to obtain a wide specificity of regions of capture. Finally, the libraries were purified using the AMPure XP bead and then amplified. The final library was quantified and certified with the Agilent High Sensitivity Kit through a bioanalyzer. Subsequently, the library was normalized and was loaded for clustering on a MiSeq Flow Cell and then sequenced with a MiSeq Instrument (Illumina).

### RNA-Seq Inspection

After the sequencing, the quality of the demultiplexed samples was verified using the software fastQC (version 0.11.5, Babraham Institute, Cambridge, United Kingdom) along with Trimmomatic (version 0.38, Usadel Lab, Aachen, Germany) (Bolger et al., 2014) to drop adapters or bad bases. Each read was mapped to the relative gene of the human reference genome version GRCh38 through the spliced transcripts alignment to a reference (STAR)RNA-seq aligner (version 2.7.3a, New York, NY, United States) (Dobin et al., 2013). Then, the htseq-count (version 0.6.1p1, European Molecular Biology Laboratory (EMBL), Heidelberg, Germany) package (Anders et al., 2015) in python was used to count the transcripts and the differentially expressed genes (DEGs) for both the comparisons were analyzed in R (version 3.6.3, R Core Team) using the Bioconductor package DESeq2 (Love et al., 2014).

### Western Blotting

Proteins extracted from all experimental groups were processed as previously described (Soundara Rajan et al., 2017). Briefly, 30 μg of proteins were resolved on SDS–PAGE gel and subsequently transferred to nitrocellulose sheets using a semidry blotting apparatus. Sheets were saturated for 120 min at room temperature in blocking buffer (1×TBS, 5% milk, 0.1% Tween- 20) and incubated overnight at 4°C in blocking buffer containing primary antibodies Bcl-2 (1:750; Santa Cruz Biotechnology, Dallas, TX, United States), Ki-67 (1:500; BioGenex, Fremont, CA, United States), Cleaved caspase-3 (1:1,000; Cell Signalling Technology, Milan, Italy), Cleaved caspase-8 (1:400; Cell Signalling Technology), p53 (1:400; Merck Millipore, Milan, Italy) and ITGA4 (1:400; Abnova, DBA, Milan, Italy). β-Actin (1:750; Santa Cruz Biotechnology) was used as a loading control. After four washes in TBS containing 0.1% Tween-20, samples were incubated for 60 min at room temperature with peroxidase-conjugated secondary antibody diluted as 1:1,000 in 1 × TBS that contained 2.5% milk and 0.1% Tween-20. Bands were visualized and quantified by the ECL method with Alliance 2.7 (UVItec Limited, Cambridge, United Kingdom).

### Statistical Analysis

To select the DEGs and reject the genes that did not differ statistically we used in DESeq2 the Welch’s test applied to the negative binomial distribution. In detail, we corrected the *p*-value in q-value using the post-hoc Benjamini–Hochberg procedure and a threshold level of 0.05. Thus, only genes whose q-value was lower than 0.05 were defined as DEGs.

Furthermore, for both MSCs-lung and MSCs-CPAM comparisons we enriched the DEGs in both the comparison with the KEGG pathways using the clusterProfiler Bioconductor package (Yu et al., 2012). We used the default statistics test and we set 0.05 as the threshold. Even in this case, we used the false discovery rate post-hoc Benjamini–Hochberg to correct the *p*-value and drop the false positive pathways.

For protein expression the experiments were performed in triplicate. All data are expressed as mean ± standard deviation. Statistical analyses were performed using the Student’s unpaired *t*-test for comparisons of two groups and one-way ANOVA followed by the Bonferroni method for comparisons of three or more groups. Values of *p* < 0.05 were considered statistically significant.

## Results

### Characterization of MSCs-Lung and MSCs-CPAM

MSCs-lung and MSCs-CPAM were successfully isolated and expanded from lung samples of both patients. As already reported they were plastic adherent, showed spindle shape morphology and high proliferative capacity, reaching ≥80% confluence in less than 5 days.

Cells were negative for CD34, CD14, CD45, and HLA-DR and positive for CD73, CD90, CD105, and HLA-I.

They did not differentiate into adipocytes or osteocytes, since lipid droplet formation or AP activity and calcium deposition was not observed.

MSCs-lung ceased to growth at P14 for both lines, while MSCs-CPAM at P19 and P16.

Moreover, they showed a dose dependent *in vitro* immunosuppressive activity on activated PBMC.

### Culture of MSCs-Lung and MSCs-CPAM

**FIGURE 1:**
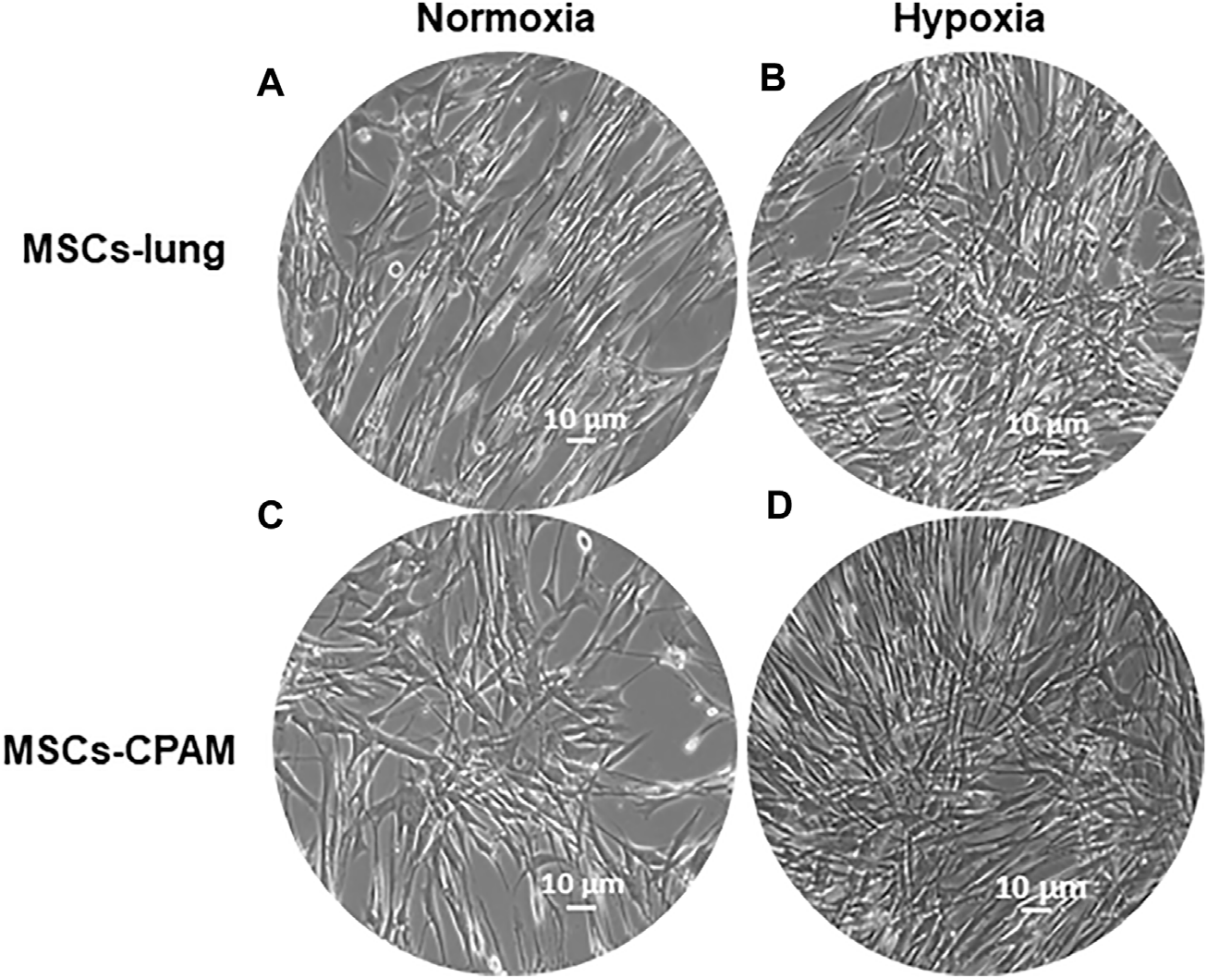
MSCs-lung and MSCs-CPAM morphology observations under inverted light microscopy. **(A,C)** Normoxic controls. **(B,D)** Hypoxia samples. Scale bar = 10 µm.

Cells were incubated under normoxic conditions and under hypoxic conditions. Cells morphology was recorded after hypoxic treatment using a light microscope. After hypoxic treatment, several spindle-shaped fibroblast-like cells were observed and their numbers evidently increased. In contrast to normoxic conditions, hypoxic conditions promoted the proliferation in MSCs-lung and MSCs-CPAM (Figure 1).

### Transcriptomic Analysis

We focus our analysis on the DEGs observed in the two cell types (MSCs-lung or MSCs-CPAM) in the hypoxia condition compared to the normoxia condition. Thus, the analyzed comparisons are:

- MSCs-lung-Normoxia against MSCs-lung-Hypoxia
- MSCs-CPAM-Normoxia against MSCs-CPAM-Hypoxia.

**FIGURE 2:**
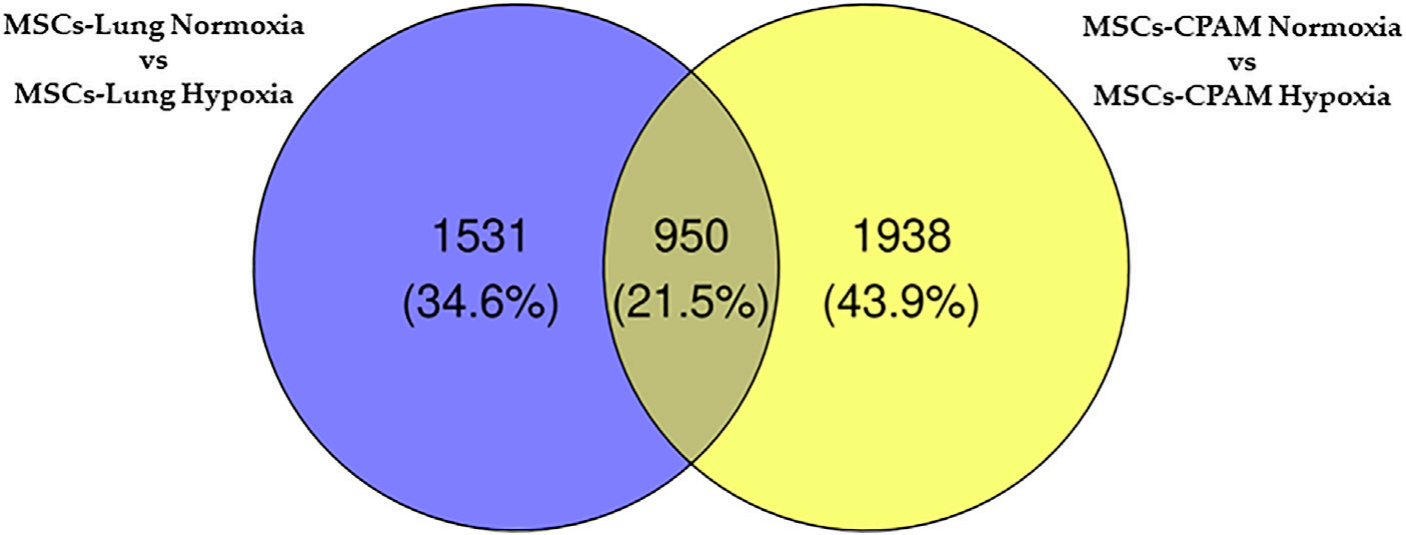
Venn diagram of MSCs-lung and MSCs-CPAM in the different normoxia and hypoxia conditions. All the comparisons found 606 genes as different in statistical manner (the bottom intersection).

The comparison of MSCs-lung-Normoxia against MSCs-lung-Hypoxia highlighted 2,481 DEGs (1,361 upregulated, 1,120 downregulated). On the other hand, the comparison of MSCs-CPAM- Normoxia against MSCs-CPAM-Hypoxia highlighted 2,888 DEGs (1,501 upregulated, 1,387 downregulated). The Venn diagram in Figure 2 summarizes the similarity and differences in DEGs among MSCs-lung and MSCs-CPAM in normoxia or hypoxia conditions.

**FIGURE 3:**
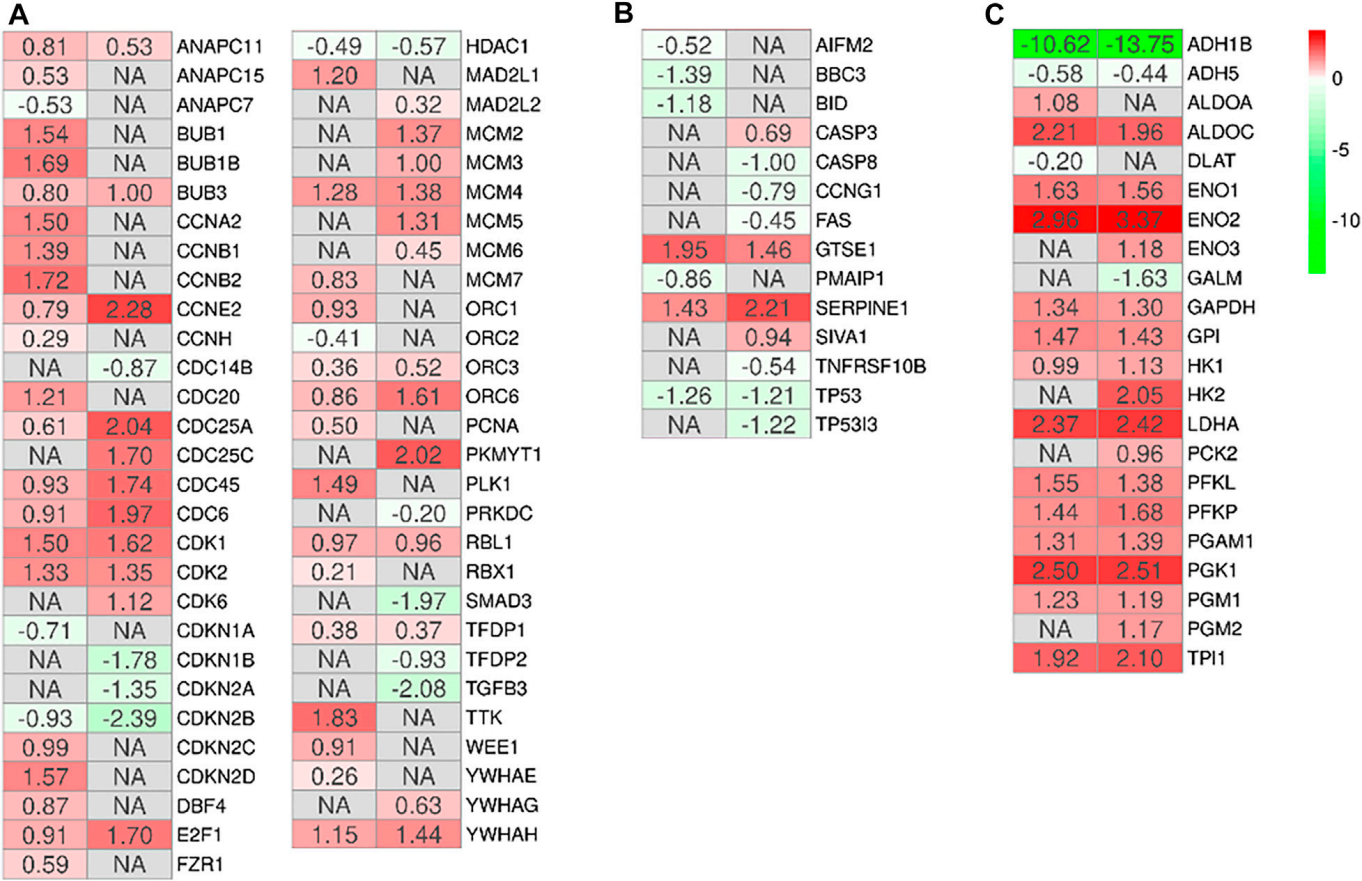
Heatmap of genes involved in the activation of the cell cycle **(A)**, inhibition of the apoptosis **(B)** or activation of the cellular metabolism **(C)** in the comparison of MSCs-lung-Normoxia against MSCs-lung-Hypoxia or MSCs-CPAM-Normoxia (left column of each comparison) against MSCs-CPAM-Hypoxia (right column of each comparison). The green scale is related to downregulated genes whereas the red palette represents upregulated ones. NA value is put when the difference is not statistically relevant in the comparison. All fold changes are rounded to the second decimal digit.

In order to observe the biological differences between MSCs-lung and MSCs-CPAM, we searched for the common enriched pathways. We focused our attention on the pathways “Cell cycle” (hsa04110), “p53 signaling pathway” (hsa04115), “Glycolysis/Gluconeogenesis” (hsa00010). The analysis of these pathways allowed us to observe 3 different processes involved in increasing the proliferative capacity of MSCs and which could support their role in tissue repair and regeneration. Indeed, treatment with hypoxia seems to increase their ability to proliferate through the activation of the cell cycle (Figure 3A) and inhibition of the apoptosis (Figure 3B), which is parallelly supported by the activation of the cellular metabolism (Figure 3C).

**FIGURE 4:**
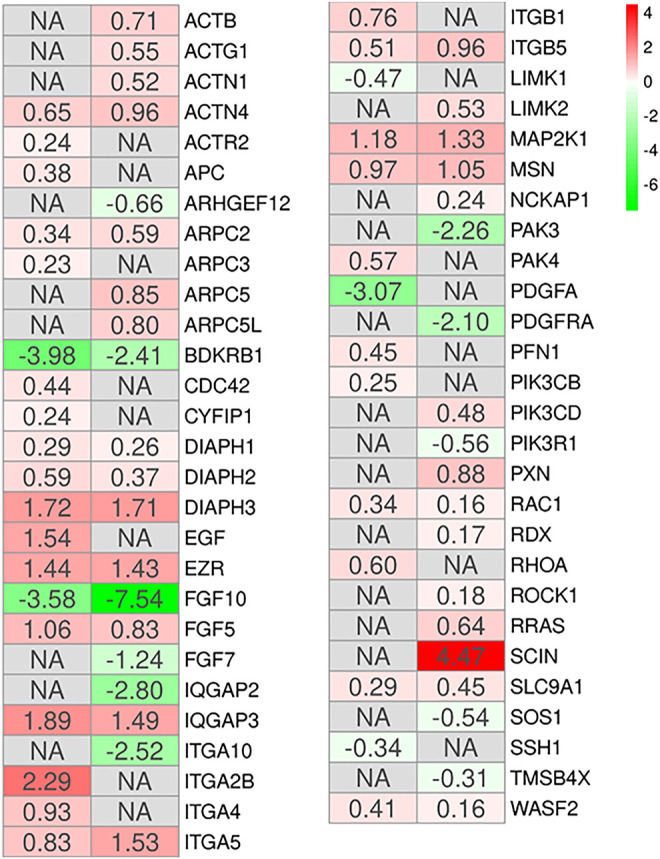
Heatmap of genes involved in the gene ontology term “Epithelium development” and observed in the KEGG pathways “Regulation of actin cytoskeleton,” “Focal adhesion” or “PI3K-Akt signaling pathway” in the comparison of MSCs-lung-Normoxia against MSCs-lung-Hypoxia (left column of each comparison) or MSCs-CPAM-Normoxia against MSCs-CPAM-Hypoxia (right column of each comparison). The green scale is related to downregulated genes whereas the red palette represents upregulated ones. NA value is put when the difference is not statistically relevant in the comparison. All fold changes are rounded to the second decimal digit.

Additionally, literature shows that the MSCs have a potential role in tissue repair (Parekkadan and Milwid, 2010). The gene ontology biological process term “Epithelium development” collects all the genes involved in the progression and formation of the epithelium to the mature structure (Attrill et al., 2019). It is known that MSCs can graft as lung epithelium in order to carry out structural repair of the damaged lung (Weiss and Finck, 2010), and the GO “epithelium development” has been identified as involved in the processes of wound healing and epithelial regeneration in other pathological conditions (Pokrywczynska et al., 2019). Thus, we enriched the KEGG pathways all DEGs of the two comparisons MSCs-lung and MSCs-CPAM included in this biological term. The pathways enriched in both the two comparison are “Regulation of actin cytoskeleton (hsa04810),” “Focal adhesion (hsa04510),” and “PI3K-Akt signaling pathway (hsa04151),” were common pathways observed. The reorganization of the cytoskeleton, the cellular adhesion and the PI3Ks signaling are pathways involved in the migratory activity and in the tissue regeneration processes (Figure 4). This consideration is in line with the cell cycle activation since and cell proliferation. Interestingly, the MSCs-lung seems more inclined to trigger the activation of these processes than the MSCs-CPAM.

### Protein Expression

**FIGURE 5:**
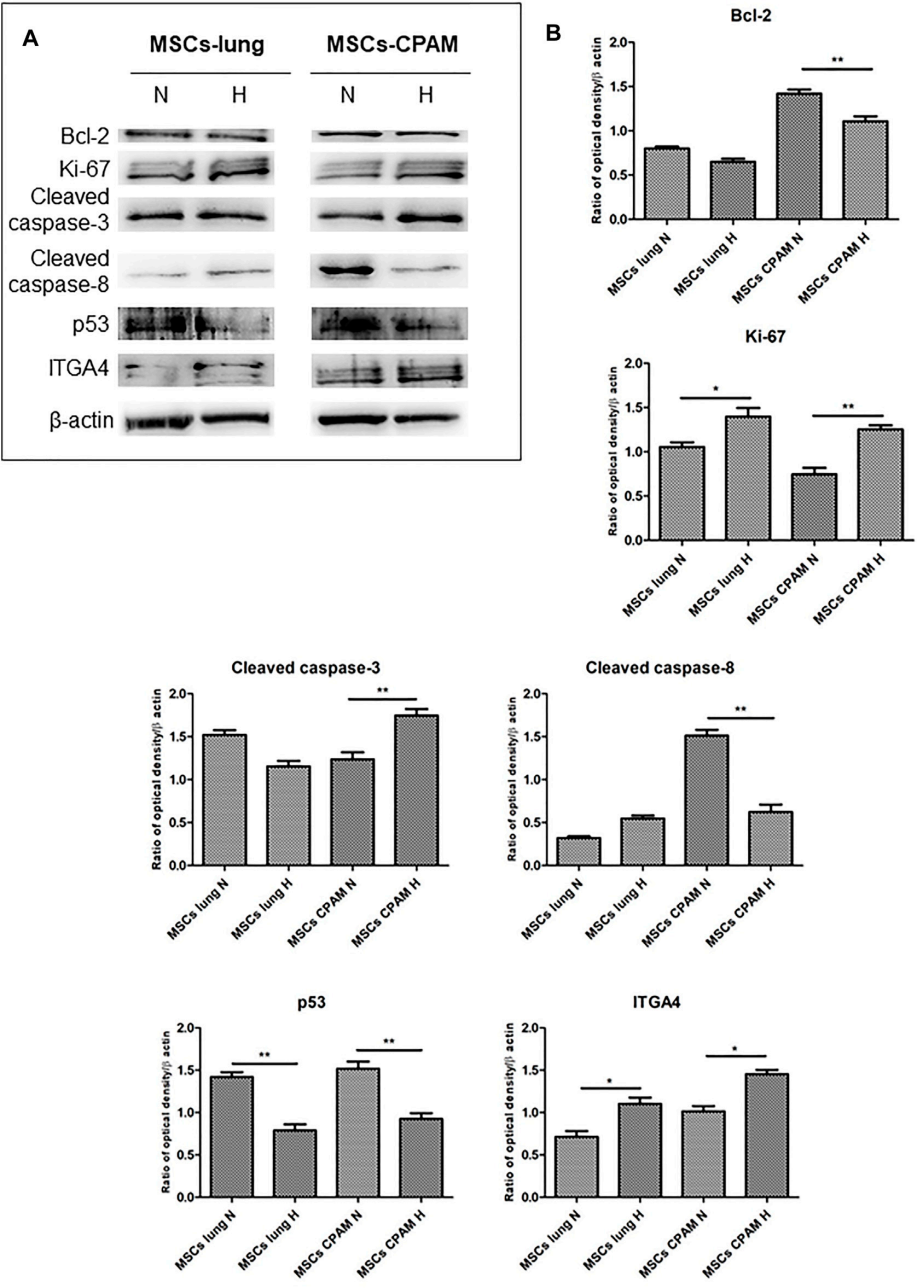
MSCs-lung and MSCs-CPAM protein expression under Normoxic (N) and Hypoxic (H) conditions. **(A)** Specific band of investigated markers Bcl-2, Ki-67, Cleaved caspase-3, Cleaved caspase-8, p53 and ITGA4. Beta-actin was used as housekeeping protein. **(B)** Densitometric analyses of the studied markers. *<*p* < 0.05; **p < 0.01.

Bcl-2, Ki-67, Cleaved caspase-3, Cleaved caspase-8, p53 and ITGA4 were investigated by Western blot analysis. The expression of Ki-67 and Cleaved caspase-3 was significantly increased in MSCs-CPAM cultured under Hypoxic conditions when compared to the MSCs-CPAM culture maintained under standard atmosphere parameters. On the other hand, Bcl-2 was down regulated in MSCs-CPAM maintained in hypoxic incubator in comparison with normoxic conditions. Cleaved caspase-8 and p53 were downregulated in MSCs-CPAM under hypoxic culture conditions when compared to the normoxic maintained culture. ITGA4 showed an increase expression in MSCs-lung and MSCs-CPAM under hypoxic conditions (Figure 5).

## Discussion

MSCs own the ability to differentiate into adipocytes, osteoblasts, chondrocytes and other tissues of mesodermal origin (Friedenstein et al., 1976; Colter et al., 2001) and possess specific immune regulatory properties (Krampera et al., 2003; Krampera et al., 2006). These properties make them interesting in cell therapy for the regeneration of damaged tissues of various origins, including epithelial tissues such as the lung. CPAM is a congenital disease characterized by cystic formations in the airways during fetal lung development and represents a model of study of congenital lung malformations which include the more complex form of pediatric interstitial lung disease. To date, surgery remains the only solution in symptomatic forms of CPAM (Leblanc et al., 2017). The regenerative process in the lung depends on a pool of lung MSCs which should allow lung regeneration with the maintenance of normal architecture during the regeneration phase (Pelizzo et al., 2017).

MSCs-based therapies represent a valid alternative for chronic lung diseases. However, it is known that MSCs, after transplantation, reduce their proliferative, survival, engraftment and paracrine properties. Several clinical studies have investigated cell therapy in chronic lung diseases (Kotton, 2012; Wecht and Rojas, 2016). The safety of MSCs therapy has only been demonstrated at an early stage and the relatively small number of recruited patients represents limitations yet to be overcome. The limited number of eligible lung donors and the potential risk of immunosuppression in patients associated with transplantation require further investigation (Weiss, 2014). Therefore, it is necessary to find strategies to improve the regenerative capacities of MSCs. The potential to enhance the benefits of MSCs has provided new opportunities that should be further explored. Indeed, the beneficial effects of hypoxic culture on proliferation and differentiation potentials of MSCs have been suggested as a strategy to improve MSCs’ properties (Grayson et al., 2007; Hung et al., 2012). As previously demonstrated (Pelizzo et al., 2017), results of the characterization of MSCs-lung and MSCs-CPAM demonstrated that the cells were adherent to the plastic and have the characteristic “spindle-shaped” morphology typical of MSCs. Moreover, hypoxic treatment did not induce morphological changes in MSCs demonstrating that it has no significant effects on the phenotype of MSCs, as already observed in other MSCs types (Valorani et al., 2012; Choi et al., 2014). Considering the involvement of hypoxia in increasing MSCs’ properties, it was performed transcriptomic analysis focused on the comparison of MSCs-lung-Normoxia against MSCs-lung-Hypoxia and MSCs-CPAM-Normoxia against MSCs-CPAM-Hypoxia, in order to evaluate the impact of 24 h of hypoxia (0.2% O2) in MSCs-CPAM compared to MSCs-lung.

First, the effects of hypoxia on proliferation and cell cycle were evaluated. It is known that to ensure cell proliferation, the cell cycle must be controlled by a regulatory network and the transition from G1 to S phase must be highly controlled. In our analysis, data suggest the existence of O_2_-dependent mechanisms that control cell proliferation. In the cell cycle, cyclins bind to cyclin-dependent kinases (CDK) in the G1 phase, then this will activate them and promote the phosphorylation of the retinoblastoma protein (Rb), with consequent release of E2F. E2F is a transcription factor that regulates the expression of proteins necessary for the transition from G1 to S (Bracken et al., 2004). Study results showed that the hypoxia conditions up-regulated *CCNE2* and *CDK2* genes in both MSCs-lung and MSCs-CPAM, *CCNA2* was up-regulated only in MSCs-lung and *CDK6* only in MSCs-CPAM. There are two classes of CDK inhibitory proteins (CDKI), p15, p16, p18, and p19, and the CDK protein/kinase inhibitory protein (CIP/KIP), composed of p21, p27, and p57 (Pavletich, 1999; Sherr and Roberts, 1999). Hypoxia reduced the expression of genes *CDKN1B*, *CDKN2A* and *CDKN2B* belonging to both classes in MSCs-CPAM; instead, it up-regulates two genes in the MSCs-lung *CDKN2C* and *CDKN2D*, and down-regulates two more *CDKN1A* and *CDKN2B*. In this way, it would appear that hypoxia facilitates the transition from G1 phase to the S phase better in MSCs-CPAM than in MSCs-lung. Indeed, *RB1*, the gene encoding Rb, was up-regulated by hypoxia only in the MSCs-CPAM comparison. The E2F1 gene, encoding the transcription factor E2F required for the transition from G1 to S were upregulated from hypoxia in both comparisons. However, the genes *TFDP1* and *TFDP2,* encoding the complex that controls the transcriptional activity of the numerous genes involved in the transition from G1 to S phase were upregulated by hypoxia only in the MSCs-lung comparison. As the G1/S transition is essential for cell cycle progression, these data demonstrate the maintenance of a high proliferative capacity in both MSCs-lung and MSCs-CPAM, as can be seen in Figure 1. In order to confirm cell proliferation, the Ki-67 Western blot investigation was performed, the results of which are presented in Figure 5. The cell proliferation antigen Ki-67 (Ki-67 or Ki67) is constitutively expressed in cells and is widely used as a marker of cell proliferation (Sobecki et al., 2016). Consistent with *in vitro* and RNA-seq investigations, Western blot analysis demonstrated that Ki-67 expression was significantly increased by hypoxia in both MSCs-CPAM and MSCs-lung (Figure 5), showing that hypoxia enhances the proliferation rate in MSCs. Moreover, it has been observed that cell cycle progression appears to be necessary for differentiation towards certain cell fates. Indeed, there appears to be a relationship between the cell cycle and cell differentiation (Jakoby and Schnittger, 2004). In particular, it has been observed that a cyclin of type D, proteins of the Rb family and CDK inhibitors, a subset of the phase transition proteins from G1 to S, are particularly involved in cell differentiation. Thus, some cell cycle proteins may regulate differentiation pathways, while simultaneously performing their cell cycle functions, as has been observed in terminal differentiation in the mammary epithelium (Caldon et al., 2010). In this context, recent evidence suggests that the tumor suppressor p53, in addition to the classical oncosuppressive activity, seems to have a role in the regulation of differentiation and development (Molchadsky et al., 2010). p53 regulates DNA repair, cell cycle, apoptosis, and senescence and cell proliferation (Liu et al., 2015b). Interestingly, in our analysis, the *TP53* gene that encodes for p53 was downregulated by hypoxia in both comparisons. Instead, the *CDKN1A* gene that encodes for p21, the main p53 target gene was downregulated only in MSCs-lung. Evidence of a significant decrease in p53 expression in both comparisons in hypoxic conditions was also confirmed at the protein level by Western analysis (Figure 4), suggesting that hypoxia ameliorates proliferation in both comparisons.

The results obtained show that hypoxia, in addition to stimulating the downstream genes to mediate the proliferation process, seems to deregulate the expression of the genes involved in the apoptosis process (Figure 3B). It is known that hypoxia preconditioning enhances the survival of MSCs, indeed it was observed that BMSCs in ischaemic tissues increased autophagy and decreased apoptosis, suggesting that hypoxia may result in a protective effect in MSCs. Hypoxia condition induces a similar effect also in BMSC survival *in vivo* (Liu et al., 2015a). Several findings demonstrated that 1–2% O_2_ enhances the proliferation rate of human adipose-derived mesenchymal stem cells (Fotia et al., 2015; Wan Safwani et al., 2016). These evidences indicate that more deep investigations are necessary in order to improve stem cell function under stressed or pathological conditions. In this regard, our study results showed that hypoxia, in addition to reducing the pro-apoptotic *TP53* gene in both MSCs-lung and MSCs-CPAM, increases the levels of factors that negatively regulate the apoptosis processes, such as *SERPINE1* and *GTSE1,* in both comparisons. The overexpression of *SERPINE1* would appear to protect cells from apoptosis and increase cell migration while *GTSE1* acts as a negative p53 regulator (Monte et al., 2004; Pavón et al., 2015). In addition, in MSCs-lung are highlighted down-regulated genes *BBC3*, *BID* and *PMAIP1*, known for their pro-apoptotic activity. *BBC3* and *PMAIP1* belong to the pro-apoptotic class BH3 and are part of the apoptotic cascade mediated by p53, and *BID* is a mediator of mitochondrial damage induced by CASP8 and determines the release of cytochrome c (Westphal et al., 2011; Hikisz and Kiliańska, 2012). In compliance with the RNA-seq analysis, Western blot and statistical analyses also showed a significant increase in the expression of cleaved-caspase 3 in MSCs-CPAM grown under hypoxic conditions more than in MSCs-CPAM maintained in standard atmospheric parameters. Contrarily, no significant difference was observed in the comparison of MSCs-lung under normoxic conditions compared to MSCs-lung under hypoxic conditions (Figure 5). This caspase is responsible for most of the proteolysis during apoptosis and the detection of cleaved caspase-3 is therefore considered a marker of apoptosis (Poreba et al., 2013). Therefore it would appear that hypoxia better protects MSCs-lungs from hypoxia than MSCs-CPAMs. Instead, in MSCs-CPAM, even though the *CASP3* gene was overexpressed by hypoxia, other apoptotic genes such as *CASP8*, *FAS* and *TNFRSF10B* were downregulated. *CASP8* encodes the Caspase 8 protein, a member of the cysteine-aspartic acid protease family, which is important in the execution phase of cellular apoptosis (Kruidering and Evan, 2000). In compliance with the transcriptomic analysis, also the Western analysis showed a significant reduction of the protein expression levels of cleaved-caspase-8 in MSCs-CPAM subjected to hypoxic conditions (Figure 5). *FAS* is a gene that codes for a member of the TNF receptor superfamily, involved in the regulation of programmed cell death. The interaction of this receptor induces the formation of a death-inducing signaling complex that includes Fas-associated death domain protein and caspase 8 (Waring and Mullbacher, 1999). *TNFRSF10B* encodes TNF Receptor Superfamily Member 10b, a receptor transduces an apoptosis signal (Zhao et al., 2014). Thus the downregulation of these pro-apoptotic genes and upregulation of those anti-apoptotic seems to protect more MSCs-lung than MSCs-CPAM from apoptosis. Further studies are needed to deepen the effect of hypoxia on the apoptotic process in MSCs-CPAM.

Stem cells constantly encounter hypoxic stress that hinders aerobic metabolism. Therefore, the upregulation of genes in the “Glycolysis/Gluconeogenesis” pathway under hypoxic compared to normoxic conditions, in both cell types, suggest that anaerobic metabolism was increased. In detail, *ALDOA* and *ALDOC* encode for two aldolase isoenzymes (A and C). They are two glycolytic enzymes that catalyze the reversible conversion of fructose-1,6-bisphosphate into glyceraldehyde 3-phosphate and dihydroxyacetone phosphate. In this analysis, hypoxia upregulated both genes in MSCs-lung, while in MSCs-CPAM only the *ALDOC* gene was upregulated. Hypoxia also upregulated *ENO1*, *ENO2*, and *ENO3* in MSCs-CPAM, while in MSCs-lung it upregulates only *ENO1* and *ENO2*. In mammals, three genes encoding for three isoforms of the enzyme, α-enolase (ENOA), γ-enolase and β-enolase, respectively (Capello et al., 2011). In the second half of the glycolytic pathway, these enzymes promote the dehydration of 2-phospho-D-glycerate to phosphoenolpyruvate; conversely, in gluconeogenesis, these enzymes catalyze the hydration of phosphoenolpyruvate to 2-phospho-D-glycerate (Pancholi, 2001). *GP1*, *GAPDH* and *LDHA* are hypoxia-upregulated genes in both MSCs-lung and MSCs-CPAM. The *GP1* gene encodes the glucose-6-phosphate isomerase, responsible for the conversion of glucose-6-phosphate to fructose-6-phosphate, the second phase of glycolysis, and for the reverse reaction during gluconeogenesis (Kugler and Lakomek, 2000). Furthermore, in addition to its main role as a glycolytic enzyme, it can act as an angiogenic factor by promoting the motility of endothelial cells (Funasaka et al., 2001). *GAPDH* encodes glyceraldehyde-3-phosphate dehydrogenase, an enzyme important in glycolysis that promotes the conversion of D-glyceraldehyde 3-phosphate to 3-phospho-D-glyceroyl phosphate (Sirover, 2021). Furthermore, it has been observed that this enzyme also modulates the organization and assembly of the cytoskeleton (Schmitz and Bereiter-Hahn, 2002). *LDHA* encodes lactate dehydrogenase A, involved in the conversion of pyruvate into lactic acid during the last phase of glycolysis. Additionally, increased *LDHA* expression is required for glycolysis maintenance (Zheng et al., 2021). Therefore, the upregulation of the genes that code for glycolytic enzymes and the increase of the gene that codes for Lactate Dehydrogenase, make both MSCs-lung and MSCs-CPAM more dependent on anaerobic glycolysis for energy supply. These results would highlight the ability of hypoxia to facilitate the metabolic transition necessary to support the energy demands. Thus, the MSCs subjected to hypoxic challenges, increased genes encoding the glucose-6-phosphatase transporter, lactate dehydrogenase-A, glycolytic enzymes and glucose transporters to facilitate the glycolytic pathway. In this way, the modulation of glucose metabolism in MSCs, together with the down-regulation of anti-apoptotic factors represent a strategy to protect MSCs from apoptosis, improving their viability in hypoxic conditions (Hu et al., 2008).

Several studies have shown that MSCs in hypoxic conditions have a high regenerative potential. It has been seen that hypoxia increase cell proliferation, survival in damaged tissues after transplantation, and secretion of several bioactive factors (Noronha et al., 2019). Short-term hypoxic exposures induce functional changes in MSCs, such as alteration of glycolysis, reorganization of the cytoskeleton, and increased migratory activity (Zhidkova et al., 2021). In addition, mesenchymal-epithelial interaction plays an essential role in regeneration processes where it contributes to the maintenance of tissue homeostasis and its repair against damage (Demayo et al., 2002).

In this context, we inspected DEGs involved in epithelium development. Among all the pathways found in the two comparisons, “Regulation of actin cytoskeleton,” “Focal adhesion,” and “PI3K-Akt signaling pathway,” were common pathways observed (Figure 4). This result is in compliance with the cell cycle activation given the involvement of the regulation of the cytoskeleton in cell proliferation (Bendris et al., 2015). The actin cytoskeleton is known to regulate cell adhesion and motility through its intricate participation in signal transduction and structural modifications. In both comparisons the hypoxic condition, upregulating many of the genes involved in this pathway, could reorganize the cytoskeleton thus influencing the processes necessary for the regulation of epithelia.

Among these genes, *RHOA* appears upregulated in MSCs-lung under hypoxic conditions compared to MSCs-lung in normoxic conditions. While *RAC1* appears upregulated in hypoxic conditions compared to normoxia in both MSCs-lung and MSC-CPAM, *CDC42* instead appears upregulated only in MSCs-lung in hypoxic conditions compared to MSCs-lung-Normoxia. These genes encode for members of the Rho family of small guanosine triphosphatases (GTPases), involved in the regulation of actin remodeling, adhesion site formation, and actomyosin contraction (Hall, 1998; Nobes and Hall, 1999). *RAC1* is involved in the formation of focal complexes and the formation of lamellipods through the polymerization of actin, while *CDC42* is mainly involved in cell polarity and phyllopod formation and transmits environmental signals to effector proteins, setting the orientation of the cell (Hanna and El-Sibai, 2013). The RHOA signaling cascade is believed to play an essential role in the migration of MSCs. Vertelov et al. found that in MSCs hypoxic conditions develop greater motility than normoxia in relation to *RHOA* activation hypoxia-induced, suggesting that elevated MSCs migration may occur through increased activation of *RHOA* (Vertelov et al., 2013). Therefore, hypoxia in MSCs-lung cells, through the upregulation of *RHOA* and *CDC42*, could increase the regulation of cell adhesion and migration processes.

*RHOA* activation occurs through a variety of factors such as growth factors, cytokines, adhesion molecules, integrins, G proteins and other biologically active substances (Loirand et al., 2006). During repair processes, components within the cellular matrix interact with integrins (Chen and Parks, 2009). Integrins are heterodimeric transmembrane glycoproteins receptors formed by non-covalently associated α and β subunits. It has been observed that hypoxic conditions, as well as promoting MSCs migration, self-renewal and delaying senescence, also induce an increase in subunits α1, α3, α5, α6, α11, αv, β1, and β3 (Saller et al., 2012). In our analysis, many integrin-coding genes were more up-regulated by hypoxia in MSCs-lung than in MSCs-CPAM. In detail, *ITGA5* and *ITGB5* are up-regulated in both comparisons, while *ITGA10* was up-regulated only in MSCs-CPAM. In MSCs-lung, instead, hypoxia upregulated *ITGA2B*, *ITGA4,* and *ITGB1*. In compliance with the RNA-seq analysis, Western blot and statistical analyzes showed a significant increase in the expression of ITGA4 in MSCs-lung. However, evidence of a significant enhancement in ITGA4 expression was also observed in MSCs-CPAM under hypoxia conditions compared to MSCs-CPAM in normoxic conditions, suggesting that hypoxia could improve MSCs properties. Indeed, it is known that the expression of ITGA4 in MSC mediates cellular adhesion and cell migration (Ocansey et al., 2020). In particular, the formation of heterodimers consisting of ITGB1 and ITGA4 increases the homing properties of MSCs (Kwon et al., 2018). Therefore, overexpression of integrins, especially in MSCs-lung, could promote the epithelial repair process.

Phosphatidylinositol 3-kinases (PI3Ks) are the well-known regulators of cell motility. In this study, hypoxia has induced the deregulation of the genes encoding for PI3Ks, in particular in MSCs-lung *PIK3CB* was up-regulated, while in MSCs-CPAM *PIK3CD* was up-regulated. However, in MSCs-CPAM the upregulation of this gene was counteracted by the downregulation of the *PIK3R1* gene. The PI3Ks are involved in cell proliferation, cell transformation, paracrine function and angiogenesis (Zhang et al., 2014). It is known that the PI3K/AKT pathway plays an important role in cell proliferation induced by hypoxia (Sheng et al., 2017). In compliance with this finding, hypoxia upregulated PI3K in MSCs-lung more than in MSCs-CPAM.

**FIGURE 6:**
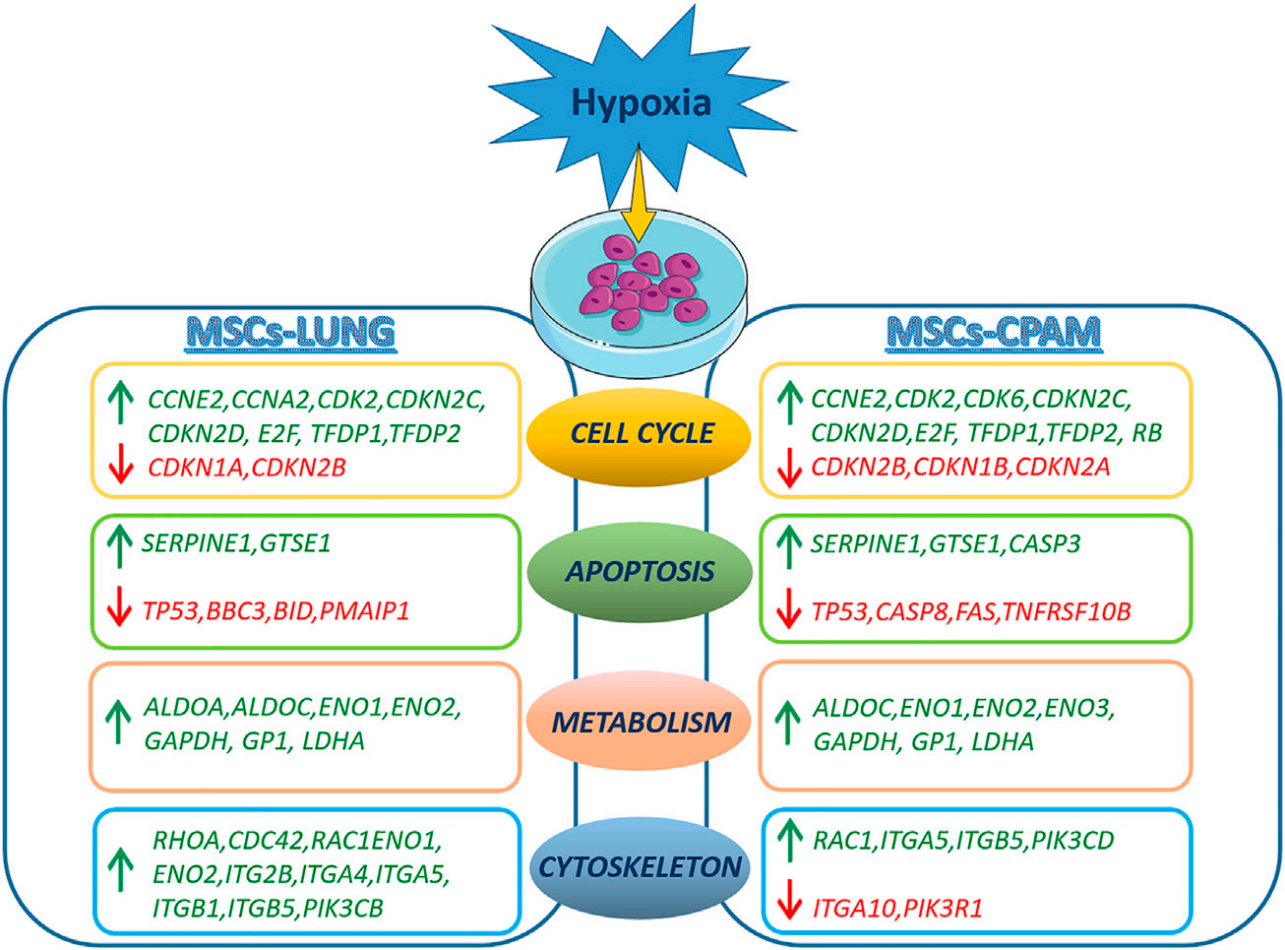
Representation of DEGs observed in the MSCs-lung or MSCs-CPAM in hypoxia condition. The figure was made taking the images from Servier Medical Art (available at http://smart.servier.com/accessed on 20 January 2022), licensed under a Creative Commons Attribution 3.0 Unported License (https://creativecommons.org/licenses/by/3.0/accessed on 20 January 2022).

In conclusion, hypoxia treatment results in cell cycle activation with increased proliferative capacity and increased cell anaerobic metabolism in both MSCs-lung and MSCs-CPAM. The analysis suggests a reduction in the apoptotic process in both comparisons, although two pro-apoptotic genes were observed up-regulated in MSCs-CPAM. Finally, data obtained indicate that hypoxia leads to a greater expression of more factors involved in cell motility, proliferation and cell migration in MSCs-lung than MSCs-CPAM (Figure 6). Therefore, the study data highlights that MSCs-CPAMs like MSCs-lungs can be isolated and expanded *in vitro* and exhibit morphology, phenotype and characteristics typical of MSCs. Noteworthy, these results suggest that exposing MSCs to hypoxia could be considered an innovative approach to lung repair and regeneration during disease progression and/or as post-surgical lung support after lung resection and encourages future studies to evaluate the importance of lung-derived MSCs in normal and pathological conditions.

## Data Availability

The datasets presented in this study can be found in online repositories. The names of the repository/repositories and accession number(s) can be found below: https://www.ncbi.nlm.nih.gov/, PRJNA800623, https://www.ncbi.nlm.nih.gov/, PRJNA752960.
